# Conformational States of Exchange Protein Directly Activated by cAMP (EPAC1) Revealed by Ensemble Modeling and Integrative Structural Biology

**DOI:** 10.3390/cells9010035

**Published:** 2019-12-21

**Authors:** Mark Andrew White, Tamara Tsalkova, Fang C. Mei, Xiaodong Cheng

**Affiliations:** 1 Sealy Center for Structural Biology and Molecular Biophysics, The University of Texas Medical Branch, Galveston, TX 77555, USA; 2 Department of Biochemistry and Molecular Biology, The University of Texas Medical Branch, Galveston, TX 77555, USA; 3 Department of Pharmacology and Toxicology, The University of Texas Medical Branch, Galveston, TX 77555, USA; tsalkovat@yahoo.com; 4 Department of Integrative Biology & Pharmacology, University of Texas Health Science Center at Houston, Houston, TX 77030, USA; Fang.c.mei@uth.tmc.edu; 5 Texas Therapeutics Institute, Institute of Molecular Medicine, University of Texas Health Science Center at Houston, Houston, TX 77030, USA

**Keywords:** EPAC1, EPAC2, Small-Angle X-ray Scattering (SAXS), guanine nucleotide exchange factor

## Abstract

Exchange proteins directly activated by cAMP (EPAC1 and EPAC2) are important allosteric regulators of cAMP-mediated signal transduction pathways. To understand the molecular mechanism of EPAC activation, we performed detailed Small-Angle X-ray Scattering (SAXS) analysis of EPAC1 in its apo (inactive), cAMP-bound, and effector (Rap1b)-bound states. Our study demonstrates that we can model the solution structures of EPAC1 in each state using ensemble analysis and homology models derived from the crystal structures of EPAC2. The *N*-terminal domain of EPAC1, which is not conserved between EPAC1 and EPAC2, appears folded and interacts specifically with another component of EPAC1 in each state. The apo-EPAC1 state is a dynamic mixture of a compact (Rg = 32.9 Å, 86%) and a more extended (Rg = 38.5 Å, 13%) conformation. The cAMP-bound form of EPAC1 in the absence of Rap1 forms a dimer in solution; but its molecular structure is still compatible with the active EPAC1 conformation of the ternary complex model with cAMP and Rap1. Herein, we show that SAXS can elucidate the conformational states of EPAC1 activation as it proceeds from the compact, inactive apo conformation through a previously unknown intermediate-state, to the extended cAMP-bound form, and then binds to its effector (Rap1b) in a ternary complex.

## 1. Introduction

**Figure 1:**
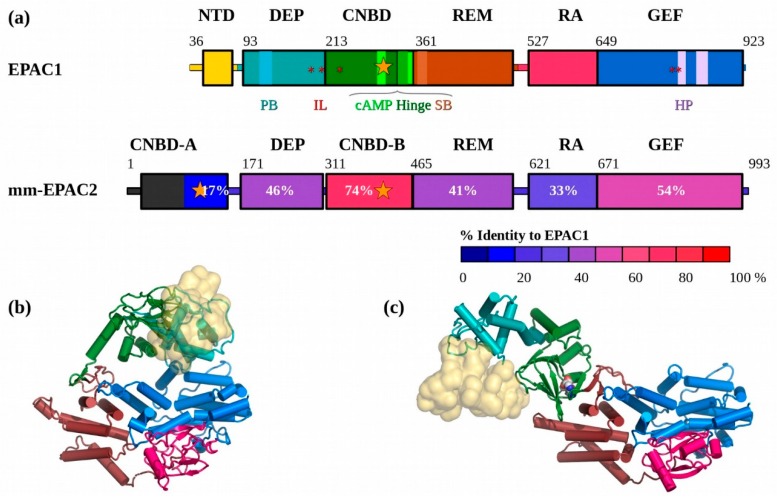
Domain structure of the EPACs. (**a**) Top, the EPAC1 domains are shown coloured: *N*-terminal domain (NTD) (CNBD-A in EPAC2), **yellow**; dishevelled Egl Pleckstrin (DEP), **teal**; CNBD (CNBD-B), **green**; RAS exchange Motif (REM), **brown**; RAS association (RA), **red**; guanine nucleotide exchange factor (GEF), **blue**. Below is the mmEPAC2 sequence coloured by homology (% identity) to EPAC1. EPAC1 lacks cAMP binding in its NTD domain. The other elements highlighted in the EPAC1 sequence are conserved: PB: Polybasic lipid binding loop (**cyan**), IL: Ionic latch residues (red *), cAMP: cAMP binding site (**bright green, orange** ★), SB: The “switchboard region” (**light shading**) which includes the F342 (F435 in EPAC2) Hinge helix (**med green**), and the HP: Helical hairpin (**mauve**). (**b**) The apo-EPAC1 homology model based on EPAC2. (**c**) The cAMP-bound EPAC1 homology model, showing the rotation of the NTD, DEP, and CNBD domains away from the catalytic domains. The cAMP molecule is shown as space-filling spheres. Models are coloured by domain using the same scheme as shown in (**a**). The low-homology NTD is shown as a semi-transparent yellow surface for reference.

In vertebrates, the majority of the intracellular functions of the pleiotropic second messenger cAMP are mediated by the exchange proteins directly activated by cAMP (EPACs) and cAMP-dependent protein kinase (PKA). EPAC and PKA share a homologous cyclic nucleotide binding domain (CNBD) [1]. Mammalian EPACs are found as two major isoforms, EPAC1 and EPAC2, which share significant sequence and structural homology, differing mostly via an *N*-terminal truncation of the first cAMP domain (CNBD-A) in EPAC1 (Figure 1) [2,3]. While purified recombinant EPAC1 and 2 proteins have similar biochemical activities in vitro, in terms of their abilities to response to cAMP stimulation and to activate down-stream effectors Rap1 and Rap2, their physiological functions are largely non-redundant due to their distinct tissue and cellular distributions and abilities to form discrete signalosomes through interaction with specific cellular partners [4]. In fact, the *N*-terminal sequence variation between EPAC1 and EPAC2 plays an important role in their functional diversities. For example, the CNBD-A of EPAC2, while very poor at binding cAMP, is critical for directing EPAC2 to the granule sites in β-cells [5]. Removal of the CNBD-A in EPAC2 alters the cellular localization of EPAC2 from the proximity of plasma membrane to the cytoplasm [6]. On the other hand, the *N*-terminal domain (NTD) of EPAC1 contains a mitochondrial targeting motif and is important for mitochondrial localization [7], as well as interaction with the ezrin–radixin–moesin (ERM) family of scaffolding proteins [8].

Since their discovery, EPAC proteins have been found to be involved in an extensive and growing number of signaling pathways [9,10]. A multitude of recent in vivo studies based on targeted gene knockout mouse models have further revealed the physiological roles of EPACs in learning and social interactions [10], cardiovascular responses [11,12,13,14], pain sensitivity in sensory neurons [15,16], as well as cellular metabolism, including glucose and lipid homeostasis [11,17,18,19,20,21,22]. These studies indicate that EPAC proteins may play an important role in the development of major diseases ranging from autism, cancer, chronic pain, and heart failure to the growing modern epidemics of obesity and diabetes [4]. Crystal structures of the Murine apo-EPAC2, the mutant apo-EPAC2-F435G, and the truncated ternary EPAC2: cAMP: Rap1b complex have been determined [23,24,25]. These structures have aided our understanding of the functional mechanisms of the EPACs. Yet, static structures, which may represent only part of a pathway, do not always reveal their working mechanism, which may be entropically driven. For example, the apo-EPAC2-F435G mutant is constitutively active, with approximately 60% of the maximal activity of WT EPAC2 being in the presence of a saturating cAMP concentration [26], but its crystal structure is little different from that of the apo-WT, which has no constitutive activity. NMR studies of the isolated cAMP-binding domain have provided valuable information about cAMP-mediated allostery and dynamics potentially important for EPAC activation [27,28,29,30,31,32]. These studies have suggested that the EPACs may exist in several distinct states or conformations: The inactive apo-EPAC may adopt two conformations, the crystallographic ‘closed’ conformation and a postulated ‘extended’ conformation [31]. The cAMP-bound EPAC may exist in a transitory compact inactive state and the crystallographically observed active ‘open’ state [24]. However, due to the molecule’s size, NMR is limited to the analysis of only isolated components of EPAC.

Small-Angle X-ray Scattering (SAXS), a technique with a long history, is particularly powerful for analyzing the conformational dynamics of biological macromolecules and their complexes (BioSAXS) [33]. Until recently BioSAXS has not had the popular success of other structural techniques, such as X-ray crystallography, NMR spectroscopy, and Cryo-Electron Microscopy. The resurgence of BioSAXS is the result of improvements in SAXS instrumentation design, particularly the new micro-source multilayer-optic coupled Kratky cameras or high-intensity synchrotron X-ray sources with 3-pinhole SAXS cameras, combined with the development of specialized software tools with which to analyze the scattering data from biological macromolecules, and to compare and refine macromolecular structure models using SAXS data [34,35,36,37]. The role of the constitutive F435G mutation in EPAC2 allosteric activation has been studied using SAXS [26]. That study has demonstrated that EPAC2 in solution exists as an equilibrium between a compact (inactive) and an extended (active) state, which shifted upon the addition of cAMP or with mutation of the Hinge F342 residue. While extensive X-ray crystallographic analyses of EPAC2 have been reported, to date, EPAC1 has been refractory to crystallization and structural determination efforts. Considering the important but non-redundant roles that EPAC1 and EPAC2 play under both physiological and pathological conditions, it is essential to comprehend the structural, dynamic, and functional differences between the EPAC isoforms. Thus, we have employed solution X-ray scattering combined with homology modeling and structure prediction [38] to study the full-length EPAC1 molecule in its apo-, cAMP-bound, and substrate-bound states. Herein, we show that SAXS can elucidate the conformational states of EPAC1 activation as it proceeds from the compact inactive apo conformation to the extended active cAMP-bound form, and finally to an effector-bound ternary complex.

## 2. Materials and Methods

### 2.1. Protein Expression and Purification

Recombinant full-length wild-type EPAC1B protein was constructed, expressed, and purified as described previously [26]. All proteins were at least 95% pure, as judged by SDS-polyacrylamide gel electrophoresis. The apo-EPAC1 samples used here were shown to be inactive without the addition of cAMP [39,40,41]. DLS measurements were performed using a Malvern Zetasizer (Malvern, UK).

### 2.2. Small-Angle X-ray Scattering (SAXS)

Samples for SAXS analysis were freshly prepared from frozen aliquots of purified EPAC1 and C-terminally truncated Rap1b (1-167) [42] in a buffer of 10 mM Tris pH 9, 1 mM EDTA, 10 mM DTT, and 500 mM NaCl. The EPAC1-cAMP complex samples were prepared by mixing EPAC1 with 1 mM cAMP, which has a reported binding affinity of 3 μM for EPAC1 [43,44]. The EPAC1: cAMP: Rap1b ternary complex was prepared by mixing EPAC1 with equimolar Rap1b. To ensure the formation of a stable ternary complex, we determined the binding between EPAC1–cAMP and Rap1 to be around 20–60 nM using Microscale Thermophoresis. These complexes were concentrated using a Centricon Plus-70 tube with a 30 kDa cut-off membrane (Millipore Sigma, Burlington, MA, USA) and then centrifuged at 50,000 g for 1 h to remove potential aggregates. All SAXS scattering data were collected on a Rigaku (Woodlands, TX, USA) BioSAXS-1000 camera, with a Rigaku FR-E++ microfocus Cu X-ray source (λ = 1.542 Å). Several 30-min frames of each sample plus both 30-min and 1-h buffer images were collected over the q-range (q = 4πsinθ/λ) of 0.012 to 0.67 Å^−1^ (Table 1). Data were processed, checked for radiation-induced aggregation, and reproducibility, before being averaged using SAXSLab (Rigaku) to produce 1D curves of the buffer and sample scattering. Differential transmission was corrected for using the SAXNS-ES web-server [45], based on the reasonable assumption that for dilute biomolecules in solution the I/σ(I) approaches zero (<1σ ) within the accessible q-range of 0.1 to 0.7 Å^−1^ (Appendix A). The server also scaled the data using a known molecular weight (MW) calibration, and the user input sample concentration C, so that Io = MW in Da. This MW calibration was determined from the sample concentration using known protein MW standards [46], which were collected periodically and then programmed into our SAXNS-ES web-server [45]. In addition, the concentration-independent method of Rambo and Tainer [47] was also used to estimate the MW.

As these experiments were performed over several months, with filament changes and optic realignments occurring during that time, the Io calibration varied between the data sets used herein. In the initial analysis the R_g_, q_min,_ D_max_, and P(r) were determined using the PRIMUS-QT program from ATSAS v2.5 [48], and the Porod molecular weight calculation was performed using the SAXSMOW webserver [51], with data to q = 0.25 (Å^−1^). Note that the Guinier radius R_g_ is related to the molecular radius of gyration, but also includes a solvation boundary layer [55]. The pair-density distribution function, P(r), shown in the figures was calculated using the iFT algorithm implemented in the BayesApp webserver [49]. The quality of the data was ascertained using the Negative Debye function, which means that the P(r) was not constrained to be positive.

Several small-angle scattering data sets of apo-EPAC1 were collected at different sample concentrations (4, 2, 1, and 0.5 mg/mL), various pH values (pH 8.7, 9.0, and 9.5), and subjected to multiple analyses R_g_ (Primus), D_max_ (GNOM), and the ensemble optimization method (EOM), following recommended procedures [56,57]. In Table 1, we report the scattering data for apo-EPAC1 with the optimal sample concentration of 0.5 mg/mL and at a pH 9.0. There were no significant differences in the SAXS curves with pH as measured by the Chi-squared metric. High concentration samples, >1 mg/mL displayed artifacts due to molecular crowding and interparticle scattering effects. Part of this was attributed to the sample preparation process, with the freshest samples, that were never concentrated beyond 0.5 mg/mL, showing the greatest fraction of the compact ensemble, lowest R_g_, and D_max_. This intrinsic sample concentration-dependent hysteresis that was also observed in EPAC2 [26], which at high concentration produces dimers, is not present in the low-concentration samples used in this study of apo-EPAC1.

### 2.3. Ab Initio Molecular Shape Analysis

Unbiased ab initio molecular shape analysis was performed using DAMMIF [50], from the ATSAS package [48]. This applies a restrained Monte-Carlo algorithm to arrange beads on a lattice so as to create a possible molecular shape that is consistent with the solution scattering data. For the cAMP-bound EPAC1 bead model, which MW calculations suggested is a dimer, we ran the refinements both with and without a two-fold (P2) symmetry imposed on the solution. In each case, 10 independent DAMMIF calculations were performed, and then filtered to create a most probable molecular shape model with DAMAVER [58], using the automated scripts available on our SAXNS website [45]. Because X-ray scattering is invariant with respect to inversions of the structure, these molecular shape models were automatically aligned with their appropriate EPAC1 homology model and corrected for any possible inversion using SUPCOMB [59] and quantified using SASREF [60].

### 2.4. EPAC1 Homology Models for Rigid-Body Analysis

Models of the EPAC1 apo-, cAMP bound-, and ternary complex were created using the same procedure as for our previous EPAC2 models used in the solution and analysis of the EPAC2-F435G crystal structure [25]. The EPAC2 crystallographic structures of the apo-EPAC2 and its ternary complex were extended using more modern crystallographic refinement and modeling tools, building into weaker density, which helped to extend the model significantly before resorting to modeling the disordered loops for which there was little clear density. For SAXS analysis, these loops must be modeled, because they contribute equally to the total scattering, even if they are disordered. The dishevelled Egl Pleckstrin (DEP) domain in the cAMP-bound and ternary complex structures was modeled based on the apo-EPAC2 structure. The initial EPAC1 models were created starting from the corresponding completed EPAC2 models using Swiss-model [61,62]. These EPAC1 models were then refined against the published Fobs for EPAC2, using PMB/CNS [63,64], and rebuilt manually in COOT [65,66]. Due to its low sequence homology, the NTD structure is uncertain. Based on Blast [67], Consurf [68], and Evolutionary Trace [69] analyses, the EPAC1 NTD is a region of low evolutionary conservation (Figure S1). Homology modeling with other tools suggested NTD domains of similar shape, but different internal structure. Since SAXS is a low-resolution technique, it is unable to resolve the internal structure of domains and therefore cannot differentiate between these models. This also means that the exact internal structure of the small NTD is not important for fitting the SAXS data, only the overall volume (Figure 1b). SAXS analyses used three possible models for the EPAC1 NTD, whose homology to the EPAC2 CNBD-A domain was low: (1) A random coil, possibly disordered; (2) a RaptorX generated computational model; and (3) a small homology domain based on EPAC2. The sequence of EPAC1 was aligned to the smallest compact beta-sheet in the EPAC2 CNBD-A, and the connecting loops modeled manually, to create a compact globular domain still in contact with the remaining CNBD. Several cycles of refinement and rebuilding were able to improve the stereochemistry of several loop regions, while maintaining the overall fold. The cAMP-bound EPAC1 homology model is derived from the ternary model by the removal of the Rap1b molecule (Figure 1c), that is assumed to cause only small and localized conformational changes upon binding which would not be observable in the SAXS curves.

### 2.5. Rigid-Body Analysis

Rigid-body analysis was performed using CORAL or Bunch from the ATSAS package [52]. Rigid-body models based on each homology model were created with a fifteen-residue linker between the NTD and the DEP domain, residues 80–94. For the cAMP–bound EPAC1 model, which MW calculations suggested was a dimer, we imposed two-fold (P2) symmetry in DAMMIF and used a dimer rigid-body model in CORAL. The NTD was modeled using three possible models: (1) A disordered chain, allowing for an intrinsically disordered protein (IDP) domain; (2) A RaptorX prediction; and (3) homology to the EPAC2 CBD-A core, as described above.

### 2.6. Polydispersity and Conformational Ensemble Analyses of EPAC1

**Figure 2:**
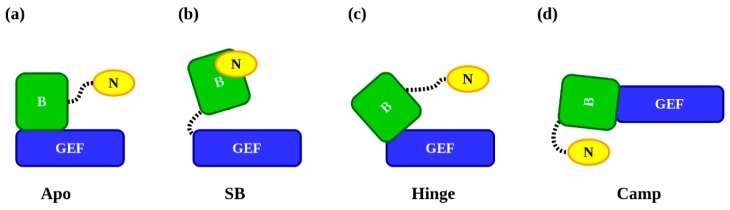
Schematic templates of EPAC1 used for ensemble generation in EOM. The dashed-lines denote flexible regions used to generate the ensembles. The NTD: “N” is in **yellow**; the regulatory DEP and CNBD domains: “B” are **green**; and the catalytic RA, REM, and GEF domains: “GEF” in **blue**. (**a**) The “Apo” model based on the apo-EPAC1 homology model with a tethered (aa 80–94) NTD. In addition, an IDP model of the NTD used a randomly generated chain-of-beads for both the linker and the NTD. (**b**) The flexible (aa 348–351) Hinge and switchboard “SB” model. This model produced four unique EPAC1_extended_ models with similar hinge angles. (**c**) These four models of the Hinge angle were used in the “Hinge” ensembles. The tethered (aa 80–94) NTD distribution is more compact than the “SB” distribution, which is under-sampled due to its high flexibility. (**d**) The “Camp” ternary-like model based on the cAMP-bound EPAC1 homology model. The Hinge and switchboard are locked and the NTD is tethered by a flexible linker (aa 80–94). For each EPAC1 EOM template, 10,000 unique models were generated.

We applied EOM [53,54] to further examine the data, to determine if the solutions were monodisperse or if EPAC1 exists as an ensemble of distinct states in equilibrium. EOM is able to distinguish between rigid and flexible proteins by considering the potential coexistence of different conformations when analyzing the experimental scattering pattern in solution. EOM is particularly powerful for studying multidomain proteins interconnected by linkers, as it directly assesses the interdomain contacts [53]. After the basic 2-body EOM analysis, using the same *N*-terminal linker, residues 80–94, as in the basic rigid-body fits, a more sophisticated multiple-ensemble 2-body analysis was performed. Several ensembles of 10,000 members each were created based on two basic scaffolds: The apo-, and cAMP-bound EPAC1 homology models (Figure 2). The apo- and cAMP–bound EPAC1 ensembles were generated from their respective homology models, each with a mobile NTD model tethered by a 15 amino acid linker, residues 80–94. These ensembles were generated for each of the three NTD models: IDP, RaptorX, and EPAC2-homology (Figure 2a). To model an intermediate state proposed by the NMR studies, a model ensemble employing a melted-Hinge and switchboard (SB) region was generated based on the cAMP-bound EPAC1 model, but with the switchboard region, residues 348–352, melted, and allowed to adopt a variety of conformations (Figure 2b). This modeled the proposed intermediate state in which the Hinge is no longer a helix, but has not yet reformed to allow part of the SB to cover the cAMP binding pocket as the lid shown in the cAMP-bound structure. EOM selected models based on four slightly different selected Hinge angles were used to create a compact pool with flexible modeling of the NTD position (Figure 2c). In a separate analysis, additional ensembles were added to represent the cAMP-bound dimer, the possibly free Rap1b molecule, and both the cAMP-bound EPAC1 monomer (Figure 2d) and the ternary complex with Rap1b. These multiple model templates generate ensembles that not only cover a broad range of R_g_ values (30–55 Å)that are accessible to the molecule, but also model the molecular shape more accurately than can a simple distribution of spheres, as employed in the McSAS algorithm [70]. Therefore, these models should capture the full distribution of shapes, finding not only the most common values of R_g_, but also the width and symmetry of such distributions, which may be due to either flexibility [54] or disorder [71]. In the analysis of overlapping distributions, we fit the frequency of R_g_ values using simple Gaussian functions for single peaks and, in the case of flat-top distributions, a double Gaussian approximation. This allows the determination of the areas of overlapping peaks. The area under each peak, calculated using the Gaussian fit, was used to determine the percent membership in each distinct conformation and the distribution peak width. Due to the variation in peaks’ shapes, the full-width at half-maximum (FWHM) values are reported. The minimal ensembles required to represent the significant members of the ensemble are presented. These tend to be more compact in their range of R_g_ values than are ensembles generated from all possible models. Using ensembles with an unnecessarily large R_g_ range reduces the resolution of the resulting distribution, since the bin width will automatically be increased to match the range. Conversely, not including models for significant members of the ensemble will skew the resulting distribution, broadening peaks and adding features not actually representative of the true ensemble [70]. The final data and models were deposited in the SASBDB [72,73], with IDs: SASDCQ6, SASDCR6, and SASDCS6.

## 3. Results

### 3.1. In Solution the apo-EPAC1 NTD Blocks the Effector Binding Site

**Figure 3:**
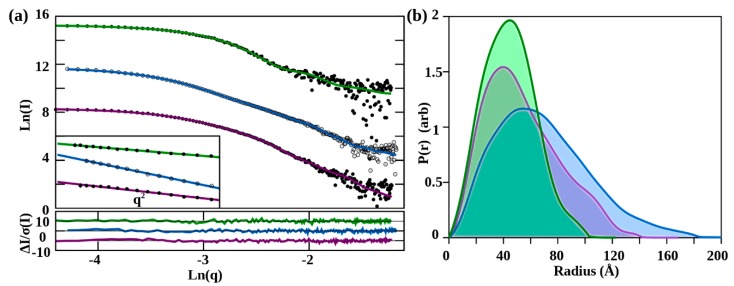
SAXS data from EPAC1. (**a**) Log–log plots of the SAXS data (**points**) with the best rigid-body model fits (**lines**) for apo-EPAC1 (**top, green lines and black dots**), cAMP bound-EPAC1 (**middle, blue lines and open circles**), and EPAC1/Rap ternary complex (**lower, purple lines and black dots**). The inset shows the Guinier plot (0 ≤ q^2^ ≤ 0.001 (Å^−1^)) for each curve, with its linear fit. The rigid-body fitting normalized-residuals are shown below the plot. (All curves are offset for clarity). (**b**) The P(r) plots. Apo-EPAC1 (**green**) D_max_ = 110 Å, cAMP-EPAC1 (**blue**) D_max_ = 185 Å, ternary-complex (**purple**) D_max_ = 142 Å.

**Figure 4:**
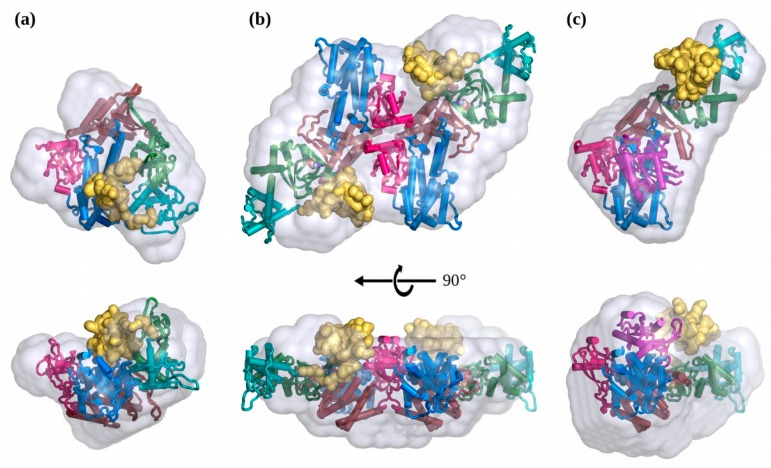
Rigid-body SAXS Models of EPAC1 in each state. (**a**) Solution structure of apo-EPAC1, in its compact state, coloured by domain as in Figure 1. (**b**) Solution structure of the cAMP bound- EPAC1 dimer, looking down the two-fold axis. (**c**) Solution structure of the EPAC1:cAMP:Rap1b ternary complex. The same domain colour scheme is used for all figures unless indicated otherwise. The NTD is displayed as a solid yellow surface, and the DAMMIF molecular shape is displayed as a semi-transparent gray surface. The catalytic GEF (**blue**), RA (**brown**), and REM (**red**) domains are in the same orientation for each model. The CORAL model SAXS curve fits are shown in Figure 3 and Table 1.

The apo-EPAC1 solution scattering data was consistent with a monomer, with a R_g_ = 33.7 Å and a D_max_ = 110 Å (Figure 3 and Figure 4a). Comparison of the scattering with the apo-EPAC1 homology model showed a reasonable fit, X^2^ = 1.3, but suggested that the single rigid-body model was not completely optimal, probably due to a fraction of the predicted extended intermediate conformation (see below). Rigid-body modeling of the apo-EPAC1 structure produced several models with similar orientations of the mobile truncated *N*-terminal domain (NTD) models, positioned over the Rap-binding site (RB) of the GEF domain, all with good fits, X^2^ ≈ 1 (Figure 3a, Table S1). The samples at different pH gave similar Rg values: pH 8.7: R_g_ = 34.6 ± 0.7 Å; pH 9.0: R_g_ = 33.7 ± 0.5 Å; pH 9.5: R_g_ = 35.0 ± 0.6 Å. The specific orientation of the NTD domain could not be determined, given both the low resolution of the SAXS data, which are insensitive to the rotation of globular domains, and the uncertain internal structure of the NTD, due to its low sequence homology. Other techniques will be needed to resolve these issues. Alternative NTD models produced identical results (Figures S2 and S3, Table S1).

### 3.2. The Solution Structure of apo-EPAC1 is a Dynamic Mixture of Closed and Extended States

**Figure 5:**
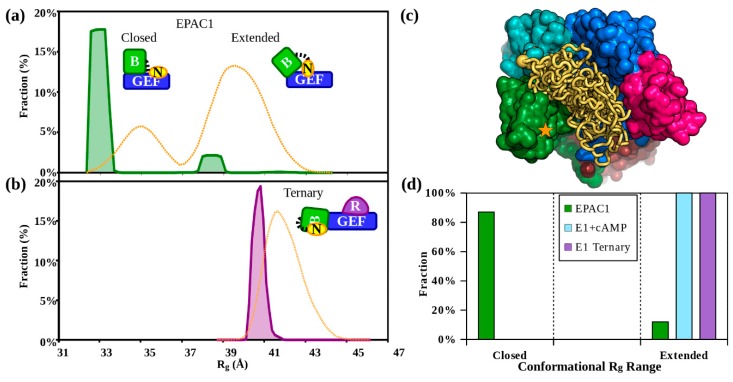
Ensemble of Models analysis of EPAC1. (**a**) EOM radius of gyration distribution analysis of apo-EPAC1 fitting (Figure S5g) and (**b**) the Rap1b-bound EPAC1 EOM R_g_ distribution, based on EPAC2 homology models. Both plots share the same x-axis scale. The apo-EPAC1 distribution is shown in solid green (**upper**) and the ternary complex in purple (**lower**). Distribution areas correspond to apo-EPAC1: Peak (EPAC1_closed_) (fraction 86%, R_g_ ~32.9 [0.8] Å), Peak (EPAC1_extended_) (13%, R_g_ ~38.5 [0.9] Å), and in the ternary complex: Peak (EPAC1_ternary_) (100%, R_g_ ~40.7 [0.7] Å). The model distribution pools generated and sampled by EOM are shown as orange dashed lines. (**c**) This EOM selected ensemble of 12 IDP NTDs (**yellow**), similarly to the CORAL models (Figures S2 and S3), block the GEF effector binding site (**blue**), but not the cAMP binding site (★) in the compact, apo-like, (EPAC1_closed_) conformation. (**d**) Histograms of the EPAC1 ensembles: apo-EPAC1, cAMP-bound EPAC1, and the EPAC1 ternary complex. The histograms are grouped by R_g_ range: Into the compact “closed” apo-conformation, or the “extended” cAMP-bound-like conformations, which includes dimer. apo-EPAC1: **green**; EPAC1 + cAMP: **blue**; EPAC1 + cAMP + Rap1b: **purple**.

The best fitting apo-EPAC1 EOM ensembles used two different models, the closed apo-EPAC1 model with a tethered NTD, plus a model of the extended conformation. Two models of the extended conformation, both based on the open structure, gave very similar results. The first, fixed the Hinge and switchboard, but allowed alternate conformations of the NTD. The second was based on the information from the NMR studies [31,32], which suggested that the Hinge melted in a fraction of the apo-EPAC1 molecules and that the SB did not form the lid of the cAMP binding site without cAMP bound to the CNBD. These studies provided an experimental basis for modeling the melted Hinge and SB region as a flexible linker. This coarse-grain ensemble resulted in a small range of selected Hinge orientations which were then used to build four compact sets of pools each with a flexible NTD. The best ensemble used two pools, the first based was on the EPAC1_closed_ model with a flexible NTD, the second the angled Hinge models with a flexible NTD. The joint EPAC1_closed_ plus Hinge distribution had two narrow peaks (Figure 5a). The EOM ensemble’s first peak, R_g_ = 32.9 Å closely matches the rigid-body model’s R_g_ of 32.7 Å. This peak represents the inactive closed apo-EPAC1 conformation (EPAC1_closed_). The (EPAC1_closed_) peak, which includes 86% of the selected models, is not a Gaussian distribution, but is flat-topped, indicating a constrained distribution about the mean with a narrow width of 0.8 Å (Figure 5). The lowest R_g_ members of the EOM generated model pool are highly over-represented in the ensemble selected by EOM, indicating that this peak represents the most compact structures (Figure S4). The second 13% (EPAC1_extended_) peak of the apo-EPAC1 distribution, with an R_g_ = 38.5 Å and a narrow FWHM of 0.9 Å, corresponds to an intermediate with a melted Hinge (Figure 2 and Figure S5), but without part of the SB forming the lid of the cAMP binding pocket, as observed in the cAMP-bound structure. This intermediate model of aop-EPAC1 is consistent with NMR studies of the isolated EPAC1 CNBD (aa 169–318) without the SB showing that EPAC1 CNBD exists in an equilibrium between a closed (~80%) and an extended conformation (~20%) in the absence of cAMP [32]. At high protein concentrations, some EOM distributions of the apo-EPAC1 show an insignificantly small peak with an intermediate R_g_ of roughly 36.0 Å. A dilution series experiment suggested that this peak was an artifact of molecular crowding and inter-particle scattering. Both of the 32.9 Å and 38.5 Å R_g_ peaks in the EOM distributions are too narrow to permit a freely mobile or intrinsically disordered NTD, suggesting that the NTD is globular and adopts a specific conformation in each state (Figure 4c and Figure S5). The ensembles based on the melted switchboard (SB), residues 347–353, a three-body model, and the dimer models did not show any significant improvement in the fit, and all had broader distributions, probably due to under sampling (Figure S4). In general, these alternative models produced similar (EPAC1_closed_) and (E_extended_) states and occupancies, but with greater peak broadening indicating that these models were not as close to the correct molecular shape or that their ensembles were too coarse due to excessive flexibility resulting in under sampling. Dynamic light scattering (DLS) analysis of this sample shows a single monodisperse peak consistent with both the (EPAC1_closed_) and (EPAC1_extended_) states, which are too close to separate (Figure S6).

### 3.3. In Solution, cAMP-Bound EPAC1 Forms Dimers

Using the EPAC1:cAMP:Rap1b ternary complex’s structure as a guide, which has a R_g_ = 40.5 Å, and then removing the Rap1b molecule yields an expected R_g_ ≈ 43 Å for the EPAC1:cAMP monomer, since the Rap1b is near to the center-of-mass. The solution scattering of the cAMP-bound EPAC1 samples all showed larger than expected R_g_ (>50 Å) and D_max_ (~150 Å) values for a monomer (Figure 3b). However, Guinier plots showed a linear dependence, over the expected range for such a large R_g_ ≈ 53 Å (Figure 3a), indicating a monodisperse sample. Molecular weight calculations indicated a MW consistent with a dimer (Table 1). The ab initio molecular shape calculations, without symmetry imposed, also suggested a compact conformation with a P2 symmetry and did not show signs of contamination with larger aggregates. Subsequent analyses were therefore performed assuming a symmetrical P2 dimer. Because there was no independent determination of the possible dimer interface, we used a P2 symmetry-constrained CORAL rigid-body refinement to produce possible models, which were then compared to the ab initio DAMMIF bead model, also with P2 symmetry imposed. Dimer models based on the crystal packing observed in the cAMP-bound EPAC2 crystal structure (3CF6) did not fit the scattering data, X^2^ > 20. Therefore, we used SAXS restrained rigid-body modeling in CORAL to find an EPAC1 dimer model. Even with the limited number of data points within the Guinier approximation q-range, given such a large complex, CORAL was able to fit the scattering data very well, with X^2^ = 1.0. This model placed the NTD adjacent to the C-terminal domains of the opposite molecule of the dimer, possibly adding to the inter-molecular contacts forming the dimer. The calculated R_g_ of the elongated active EPAC1 monomer extracted from this dimer model was R_g_ = 41.5 Å, slightly smaller than that predicted due to the position of the NTD. The CORAL rigid-body dimer model is also a very good fit to the ab initio DAMMIF molecular shape model with a normalized spatial discrepancy (NSD) value of 1.0 (Figure 4b). We also used an EOM/polydispersity analysis with monomers (apo- and holo-), dimers, and higher-order oligomers. The highest oligomers did not contribute, but there was between 5%–15% monomer in solution, indicating that the dimer is in equilibrium with a compact apo-monomer, and some contributions from molecular crowding at 2.7 mg/mL. The possible dimer interface shown in Figure 4b has not been confirmed through independent measurements, however this is not biologically relevant since in vivo the cAMP-bound EPAC1 will most likely will form a ternary complex, with Rap1 or another higher-affinity cofactor, instead of dimerizing. Indeed, the addition of stoichometric Rap1b, to the binary cAMP-bound EPAC1 shows no remaining dimers only ternary complex (Figure 4c).

### 3.4. The Solution Structure of the EPAC1:cAMP:Rap1 Ternary Complex

The EPAC1 ternary samples were very well-behaved in solution, up to at least 2 mg/mL. The Guinier plots were linear, with no sign of aggregation or other inter-particle effects (Figure 3a). The R_g_ of 40.5 Å and molecular weight estimates are both consistent with the homology model (Table 1, Figure 4c). Rigid-body modeling, with a mobile NTD, was able to produce an excellent fit, X^2^ = 1.0. The best fitting rigid-body models show the NTD near to, but not interacting with, the other regulatory domains, suggesting an ensemble of conformations may be possible. Indeed, ensemble modeling reveals a distribution (X^2^ = 0.7) with a single narrow peak centered at 40.7 Å (Figure 5b). The addition of ensemble members that modeled the disassociation of Rap1b from EPAC1, and an EPAC1 dimer, revealed no contribution from these components. This also agrees with the ab initio molecular shape calculation, which fits the model very well (NSD = 0.94), with the exclusion of the NTD (Figure 4c). The ensemble was not limited by the range of model R_g_ at higher values, indicating that choosing a globular NTD did not overly restrict the ensemble. In fact, the ensemble is highly over-represented by models at the lowest extreme of the pool, suggesting that the NTD is not a disordered domain. In addition, the narrowness of the peak suggests a specific interaction holding the NTD in a single fixed orientation relative to the other domains. The SAXS models suggest that the NTD interacts with the CNBD. The three NTD models produced identical fits and similar distributions, with the EPAC2-based homology model having the narrowest ensemble. At the limits of the resolution of the SAXS data, the three models do not limit the orientation of the NTD relative to the other domains, only placing it in close proximity to some of them, and not in an extended (mobile) orientation (Figure 5c).

## 4. Discussion

Human EPAC1 and mouse EPAC2, for which crystal structures are available, share 51% overall sequence identity. Among all domains between EPAC1 and EPAC2, the cAMP-Binding Domain B (CNBD-B) domain has the highest conservation (74%). The EPAC1 *N*-terminal domain (NTD) retains only 17% identity, and does not preserve the cAMP phosphate-binding cassette found in EPAC2 CNBD-A. The dishevelled Egl Pleckstrin (DEP), RAS exchange Motif (REM), RAS association (RA), and CDC25-homology guanine nucleotide exchange factor (GEF) domains retain 46%, 41%, 33%, and 54% identity, respectively (Figure 1 and Figure S1).

Despite this sequence similarity, EPAC1 proteins have been recalcitrant to crystallization. Thus, we have generated homology models of EPAC1 based on the two states of EPAC2 which have crystal structures: The apo-EPAC2 structures (2BYV and 4F7Z), and the truncated-EPAC2 ternary structure (3CF6). The solution structures of apo-EPAC1 and apo-EPAC2 were highly similar. The homology model of the ternary EPAC1:cAMP:Rap1b complex was able, with rigid-body modeling of a tethered NTD, to fit the scattering data within the precision of the data (X^2^ = 1.0). The positioning of the fitted NTD domain suggested that it is ordered and interacts with the other domains, possibly the CNBD. The apo-EPAC1 models position the NTD such that the cAMP binding site of the CNBD is unobstructed. So, unlike EPAC2, where the CNBD-A and CNBD-B cAMP sites block each other, the EPAC1_closed_ state may be cAMP binding competent. This is consistent with the NMR analysis of a positively-coupled inhibitor binding to the EPAC1 CNBD:cAMP in the ‘closed’ state [31].

**Figure 6:**
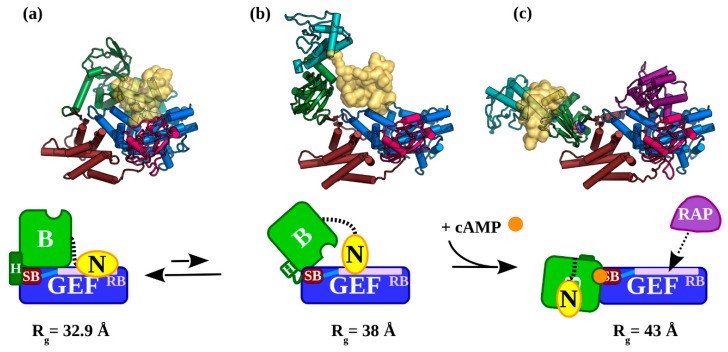
The conformational states adopted by EPAC1 during cAMP-induced activation: (**a**) Apo-EPAC1 in its inactive (EPAC1_closed_) state. The NTD, marked N (**yellow**), interacts with the catalytic GEF domain to block the effector binding site: RB (**mauve**). The Hinge (**green helix**), is fully formed and the switchboard (**SB, brown**) is held away from the cAMP binding site. The HP connecting the SB to the RB is shown in light blue. (**b**) The inactive (EPAC1_extended_) is observed with approximately a 13% frequency. This fraction of EPAC1 is receptive to activation by cAMP binding, because the Hinge has melted and the SB’s lid has not yet closed over the open cAMP binding site in the CNBD of “B”. (**c**) The active, cAMP-bound (EPAC1_open_) state of EPAC1 is capable of binding effectors such as Rap1b (**purple**) to the RB. The observed R_g_ for each state is given under each model. The atomic models are coloured as in Figure 1. The EPAC1 cartoons are coloured by region: N: NTD (**yellow**); regulatory B: DEP and CNBD (**green**); and the catalytic GEF: the REM, RA, and GEF domains (**blue**). In brown is the SB region, the Hinge (H) helix is dark green, and the RB region is mauve.

An EOM analysis of the apo-EPAC1 SAXS data showed a very narrow and compact (R_g_ = 32.9 Å) (EPAC1_closed_) ensemble. This distribution is too narrow for the NTD to be disordered. A similar tight ensemble distribution was observed for the EPAC1:cAMP:Rap1 ternary complex, suggesting that the NTD is ordered and interacts with the other domains of EPAC1 in the ternary complex. The good X^2^ of both the simple rigid-body model and the ensemble implies that the homology model of the EPAC1 ternary complex is a good approximation to its average molecular shape in solution (Figure 4c). In the inactive apo state, the ensemble fitting indicated that there was a smaller fraction of a more extended conformation, R_g_ = 38.5 Å, in equilibrium with the compact inactive closed conformation, R_g_ = 32.9 Å. This inactive extended conformation (EPAC1_extended_) is not similar to the cAMP-bound conformation seen in the ternary crystal structure or the solution dimer (Figure 4b). This intermediate (EPAC1_extended_) state is slightly more compact than the (EPAC1_open_) active state observed with cAMP bound (Figure 6), but is more extended than the inactive apo conformation (EPAC1_closed_) can accommodate, even with a disordered NTD. This could represent an inactive intermediate state observed in NMR studies, which unlike the crystallographic (EPAC2_closed_) apo-state, is competent for cAMP binding [74]. The narrow distribution peak widths of both the (EPAC1_closed_) and (EPAC1_extended_) apo states suggests that the NTD is neither intrinsically disordered nor freely rotating in solution, but rather that it is an ordered, globular domain that is interacting with other domains in a specific manner. In addition, the intermediate state is too compact to have a complete melting of the switchboard (SB) region, which would have a R_g_ significantly larger than the cAMP-bound state. This model also suggests that the NTD may continue to block effector binding until cAMP binding locks the regulatory domains away from the catalytic domains. This explains both the inactivity of the intermediate (apo) samples and the absence of any EPAC1 dimer in the ensemble analysis of samples lacking cAMP.

In the absence of Rap1, the cAMP-bound EPAC1 proteins dimerize under the conditions required for solution scattering studies. The dimer is probably an artifact of the experimental conditions, because in cells activated EPAC1 proteins are most likely associated with other binding partners such as Rap1, Rap2, ERM proteins [75] and/or the plasma membrane [76], which in vivo will most likely prevent its dimerization as is the case with the ternary complex. This notion is supported by the fact that no cAMP-bound EPAC1 dimmer can be observed in the presence of Rap1 (Table 1 and Figure 5b). Nevertheless, excepting a reorientation of the NTD to form part of the dimerization interface (Figure 4b), the conformation of the individual EPAC1 molecules within the solution dimer is not significantly different from that of the active EPAC1 conformation observed in the ternary complex (Figure 4c). These results imply a specific set of conformational states for EPAC1 (Figure 6). In the absence of cAMP and Rap1, EPAC1 is in an equilibrium of a compact apo-like (EPAC1_closed_) conformation (R_g_ = 32.9 Å), similar to that of the apo-EPAC2 crystal structure, and an elongated (EPAC1_extended_) conformation (R_g_ = 38 Å), in which the CNBD is in a cAMP-bound like conformation but without the cAMP-binding pocket covered by a lid from part of a beta-sheet of the SB. Upon cAMP binding to the intermediate (EPAC1_extended_) state, the SB move over to shield the cAMP binding site locking the regulatory NTD/DEP/CNBD away from the catalytic domains. This conformational switch allows EPAC1 to adopt the open conformation, which enables the binding of the effector without additional major structural changes to the EPAC molecule. During this process, the NTD is ordered and interacts with specific regions of the EPAC1 molecule. It is important to note that while EOM analyses can locate the general position of the NTD they are not able to provide details structural information regarding the NTD nor specific interactions between the NTD with other domains within EPAC1 as SAXS is an intrinsically low-resolution technique. Such information requires the structural determination at the atomic level.

## 5. Conclusions

Our current study provides valuable structural information about the solution conformational states of EPAC1 in its apo-autoinhibitory, active cAMP-bound, and ternary substrate complex conditions. These findings fill in a major gap in our understanding of EPAC structure and function, as very little structural information is available for EPAC1. Revelation of various conformational states of EPAC1 not only offers important structural insights into understanding of the allosteric regulation of EPAC1 activation, but also has major implications in drug discovery. It appears that inhibition of EPAC1 may be beneficial for targeting arrhythmia and heart failure [77,78], cancer [20], chronic pain [15], rickettsial infection [79], and obesity [17]. The existence of an inactive intermediate state suggests that it is possible to develop allosteric inhibitors specifically stabilizing the intermediate conformation of EPAC1 without disturbing the ubiquitous cellular cAMP signaling pathways.

## Figures and Tables

**Table 1:** SAXS Data and analysis.

	apo-EPAC1	cAMP: EPAC1	cAMP: EPAC1: Rap1b
q-range (Å^−^^1^)	0.010–0.30	0.012–0.30	0.012–0.30
Concentration (mg/mL)	0.5	2.7	1.0
Time (h)			
Sample	6	2	2.5
Buffer	6	3	11.5
Rg (Å)	33.7 ± 0.5	53.2 ± 0.7	40.5 ± 0.6
Dmax (Å)	110	185	142
MW (kDa)	100	100	119
MW_(Io)_ (kDa) {N}	104 {1.0}	178 {1.8}	98 {0.8}
MW_(Porod)_ (kDa) {N}	113 {1.1}	240 {2.4}	124 {1.0}
MW_(Rambo)_ (kDa) {N}	97.8 {1.0}	218 {2.2}	126 {1.1}
DAMMIF NSD (10)	0.8 ± 3	1.0 ± 8	0.9 ± 1
SASRES(Å)	40 ± 3	14 ± 2	42 ± 3
Rigid Body Fitting			
HM Crysol fit (X^2^)	1.3	NA	NA
CORAL fit (X^2^)	1.0	1.0	1.0
CORAL Model R_g_ (Å)	32.7	41.5 ^1^	40.5
EOM:(X^2^)	1.0	1.1	0.7
R_g_ [FWHM] ^2^ {Area}			
Peak (closed)	32.9 [0.8] ^2^ {86%}	0	0
Peak (extended)	38.5 [0.9] ^2^ {13%}	0	0
Peak (ternary)	0	0	40.7 [0.7] ^2^ {100%}
Peak (dimer)	0	51 [3] ^2^ {100%}	0
SASDB-id	SASDCQ6	SASDCR6	SASDCS6
**Analysis**	**Software:**	**Analysis**	**Software:**
Image Data-processing:	SAXSLAB (Rigaku)	Buffer Subtraction:	SAXNS-ES ^3^ [45]
Data-analysis:	PRIMUS [48]	Real Space P(r):	GNOM [48], BayesApp [49]
*Ab initio* Models:	DAMMIF [50]	Molecular Weight:	SAXSMOW [51]
Rigid Body Refinement:	CORAL [52], BUNCH	Ensemble Analysis:	EOM [53], MES [54]

^1^ Dimer R_g_ is per monomer. The Coral dimer has a Rg of 54 Å. ^2^ The peak’s full-width at half-maximum (FWHM). ^3^ Appendix A.

## Supplementary Materials

The following are available online at https://www.mdpi.com/2073-4409/9/1/35/s1, Figure S1: Sequence comparison of the EPACS; Figure S2: CORAL models of the apo-EPAC1 structure; Figure S3: CORAL models of apo-EPAC1 with an IDP NTD; Figure S4: Alternate EOM Model R_g_ Distributions; Figure S5: EOM Hinge and switchboard “SB” Ensemble Models; Figure S6: DLS analysis of apo-EPAC1; Table S1: CORAL Model Dependence; Table S2: The apo-EPAC1 EOM distributions with alternative Models.

## Appendix A

### SAXNS_ES Buffer Subtraction, MW Calibration, and MW Calculation

In order to use SAXS data, the scattering from the sample cell and solution must be removed from the measured scattering intensities. This is accomplished by measuring both a sample in solution and the solution separately, then subtracting the latter from the former. Scale factors, based on X-ray transmission factors are often applied to reduce the effects of differential X-ray absorption. However, in practice we observed changes in the extended scattering (WAXS) region that were uncorrelated with the sample. The Rigaku BioSAXS-1000 collects data over a wide q-range, up to almost q ~0.7 Å^−1^, where most dilute protein samples do not have significant signal. At high q-values (q > 0.5 Å^−1^), we expect I/σ(I)→0 so that I_sample_ ≈ I_buffer_ within the error of experimental measurement. Therefore, we used this region to estimate the optimal buffer intensity scale factor for the subtraction. The SAXNS_ES program was implemented as a web-service using PERL and HTML. The program accepts two SAXS data files; one for the sample and one for the buffer, formatted in standard free-format 3-column {q, I, σ(I)} ASCII. The scale factor for the buffer is estimated from the extended (WAXS) scattering region, using the minimum averaged I_sample_/I_buffer_ ratio, in intervals of n data points. The web interface also permits the user to enter a fixed scale factor for those cases where the extended scattering still contains strong features and averaging the above ratio would not be appropriate.

Scale = Min (Σ_i_^i+n^ I(q_i_)_sample_/Σ_i_^i+n^ I(q_i_)_buffer_), for all i, q_i_ >q_min_, q_i+n_ < = q_max_, (A1)

The buffer subtracted SAXS curve is then analyzed to determine R_g_ and D_max_, and the SAXS curve and P(r) plots are generated using gnuplot. The web-server is calibrated to scale I to a known MW standard collected locally, so that I_o_ = MW in Da, given the user-input sample concentration. In addition, the method of Rambo and Tainer [47] is used to estimate the MW of the sample, and the apparent oligomerization number N based on the known, user input, MW. The outputs of these analyses are written in the header of the buffer-subtracted SAXS curve data file which the user can download along with the plots. The URL for the SAXNS_ES web-service is https://xray.utmb.edu/saxns_es.html. This method of buffer subtraction has been observed to produce more consistent results than assuming a buffer scale of 1.000 for all cases.
