# Supplementary material for: Conformational States of Exchange Protein Directly Activated by cAMP (EPAC1) Revealed by Ensemble Modeling and Integrative Structural Biology
Source: Cells. 2019 Dec 21;9(1):35. doi: 10.3390/cells9010035 (PMC7016869; doi:10.3390/cells9010035)
Supplement: Supplementary file 1 [file cells-09-00035-s001.pdf]

Conformational States of Exchange Protein Directly Activated by cAMP (EPAC1) Revealed by Ensemble Modeling and Integrative Structural Biology

Mark Andrew White [1,2,*], Tamara Tsalkova [3], Fang C. Mei [4,5] and Xiaodong Cheng [4,5,*]

[1] Sealy Center for Structural Biology and Molecular Biophysics, The University of Texas Medical Branch, Galveston, TX 77555, USA
[2] Department of Biochemistry and Molecular Biology, The University of Texas Medical Branch, Galveston, TX 77555, USA
[3] Department of Pharmacology and Toxicology, The University of Texas Medical Branch, Galveston, TX 77555, USA
[4] Department of Integrative Biology & Pharmacology, University of Texas Health Science Center at Houston, Houston, TX 77030, USA
[5] Texas Therapeutics Institute, Institute of Molecular Medicine, University of Texas Health Science Center at Houston, Houston, TX 77030, USA
* Correspondence: Xiaodong.cheng@uth.tmc.edu; Tel.: 713-500-7487, mawhite@utmb.edu; Tel.: 409-747-4747

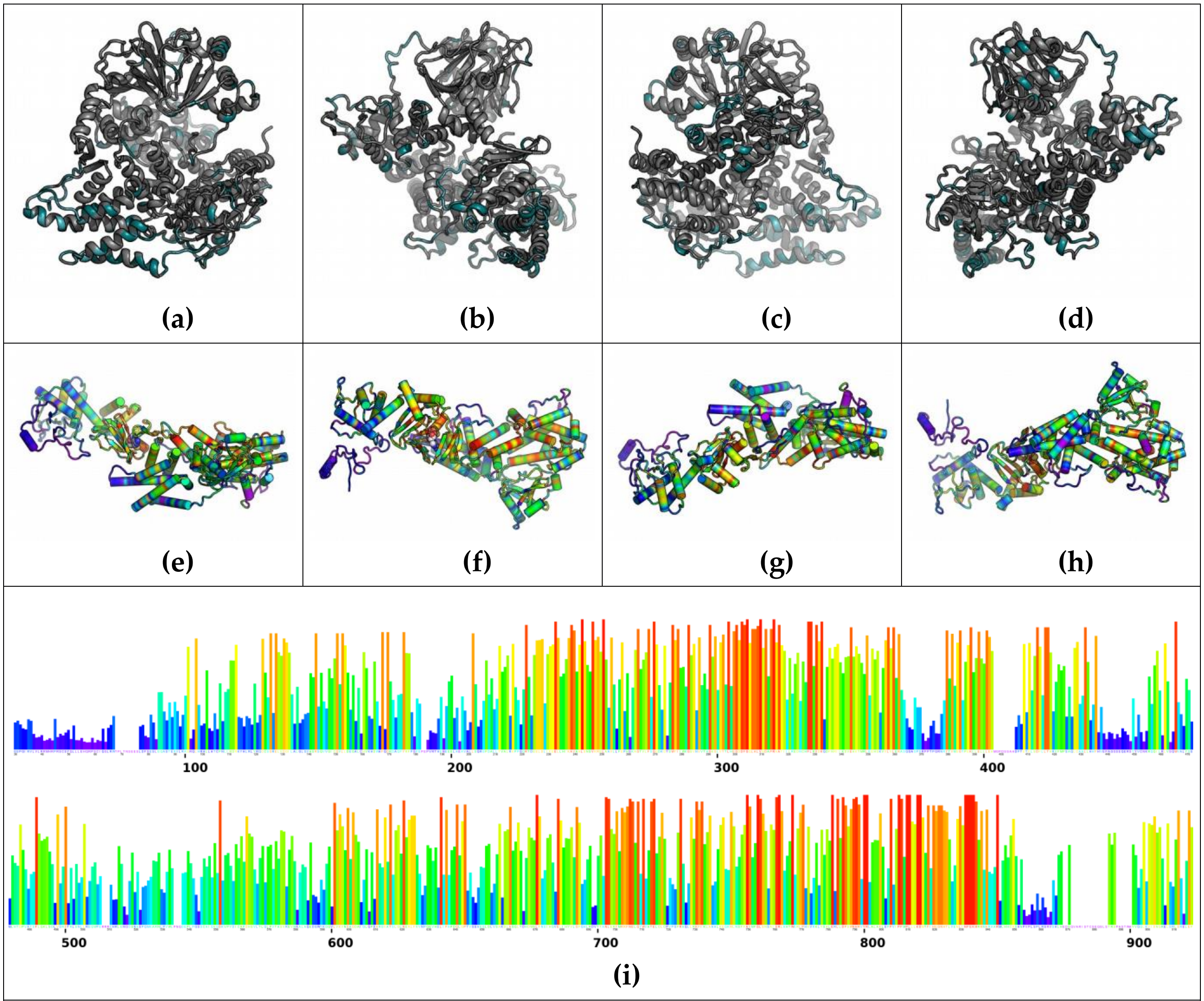

Figure S1. The Sequence Conservation in EPACs. (a, b, c, and d) The EPAC2 Consurf conservation plot showing the conserved (green) regions. Each image is rotated 90° from the previous one. (e, f, g, and h) The Evolutionary trace plots of the cAMP-bound EPAC1 model, colouring is from low-conservation (purple) to highly conserved (red). (i) The Evolutionary Trace conservation histogram. The areas with greatest conservation correspond with the cAMP binding site in the CNBD and the effector biding site in the GEF domain.

EPAC1 Homology Modeling using the SwissModel, I-TASSER, and RaptorX Servers.

CORAL Rigid Body Models

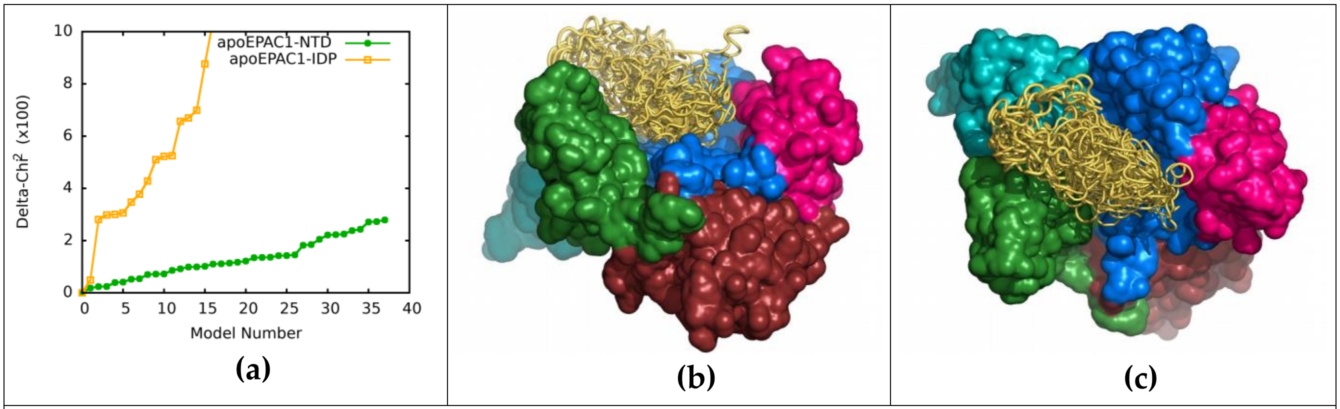

Figure S2: CORAL models of the apo-EPAC1 structure. (a) ΔChi² values of top apo-EPAC1 CORAL models. Curves are for IDP/random-coil (□ **orange/upper curve**) or globular/structured (● **green/lower curve**) NTD models. (b and c) All of the structured CORAL apo-EPAC1 models in orthogonal views, the augmented-Swiss-Model (EPAC2-homology) is shown in surface representation. **NTD**: yellow cartoon, **DEP**: teal, **NBD**: green, **REM**: brown, **RA**: magenta, **GEF**: blue.

The best apo-EPAC1 CORAL solutions all represent compact ordered conformations of the N-terminal domain, (NTD) positioned in the cleft between the regulatory and catalytic lobes (Figure S1). This combined with the narrow single distribution in the EOM analysis suggests that the NTD adopts a particular conformation in close proximity to the core of EPAC1. The best-fit models all yield equivalently good Chi² values, since the resolution of the SAXS data is insufficient to differentiate between the very similar apo-core templates or the comparable, globular, NTD templates (Table S1). The disordered (IDP) CORAL chain-of-beads refinements also each produce a globular NTD positioned in the same cleft as the other models (Figure S2).

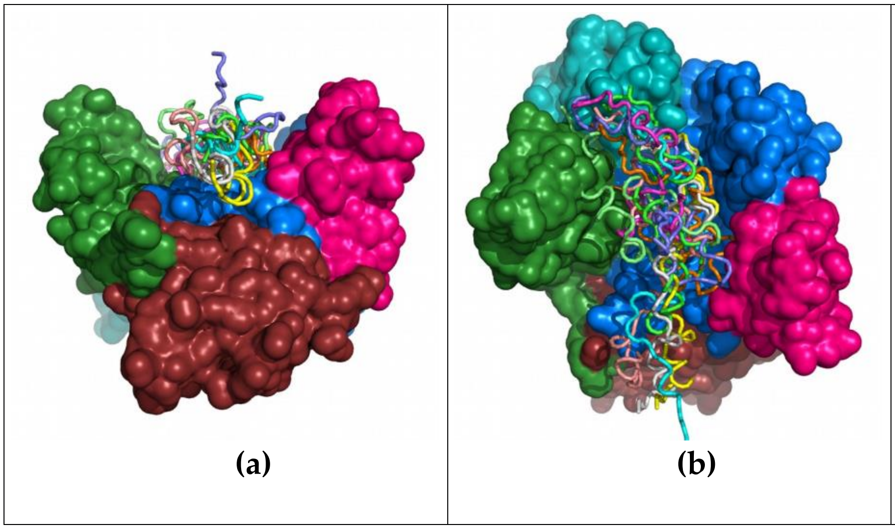

Figure S3: CORAL models of the apo-EPAC1 structure with an IDP NTD. (a and b) All of the 'disordered' CORAL apo-EPAC1 models in orthogonal views. The apo-core is shown in surface representation, the NTD is represented as a backbone-tube. **NTD**: various colours, **DEP**: teal, **NBD**: green, **REM**: brown, **RA**: magenta, **GEF**: blue.

Table S1. CORAL results for apo-EPAC1, with various core and NTD models. SM_# = Swiss Model #, IT-# = ITasser model #, Aug-SM is the manually curated SwissModel, Random indicates the Ca bead model used by CORAL to model linkers.

core\NTD	Aug-SM	IT-1	IT-2	IT-3	IT-4	IT-5	RaptorX	IDP
**Aug-SM**	1.11	-	-	-	-	-	1.11	1.12
**IT-1**	1.12	1.12	1.11	1.12	1.11	1.11	1.11	1.14
**IT-2**	1.13	1.13	1.13	1.13	1.13	1.13	1.12	1.17
**IT-3**	1.11	1.12	1.12	1.12	1.11	1.12	1.11	1.13
**IT-4**	1.11	1.11	1.11	1.11	1.12	1.11	1.10	1.12
**IT-5**	1.12	1.13	1.12	1.12	1.13	1.12	1.12	1.14
**SM_01**	-	-	-	-	-	-	-	1.15
**SM_02**	-	-	-	-	-	-	-	1.10
**SM_03**	-	-	-	-	-	-	-	1.21

SASBDB Depositions

The following EPAC SAXS data files and analyses are available from the SASBDB.

apo-EPAC1: https://www.sasbdb.org/data/SASDCQ6/fideifuudc/
cAMP-EPAC1: https://www.sasbdb.org/data/SASDCR6/3dkcd3whhj/
cAMP-EPAC1:Rap1b: https://www.sasbdb.org/data/SASDCS6/ng7ptos0xv/
apo-EPAC2 WT: https://www.sasbdb.org/data/SASDH62/tscd5hc32s/
apo-EPAC2 F435G: https://www.sasbdb.org/data/SASDH72/fkr877bepx/
apo-EPAC2 F435W: https://www.sasbdb.org/data/SASDH82/h1vaaw7fcr/

APO-EPAC1 RESULTS

The Choice of Suitable Ensemble Models for Polydispersity Analysis

The choice of model used to create an ensemble determines how well the ensemble can fit the data. Broad ensemble peaks indicate a large number of models (parameters) are needed, while extremely narrow peaks indicate a single conformation (per peak) is sufficient. In the extreme case, and ensemble of spheres of varying radii used to fit data from an ideal ellipsoid will select a continuum of spheres with radii from the minimum dimension R1 to the maximum dimension R2 = D_{max}, whereas a single ellipsoid of radii (R1 and R2) will fit the data perfectly. Therefore, the peak FWHM is a good indicator of a well determined model, while distributions limited by the model pool are indications of a potential problem. In addition to the "Apo" tethered NTD model and "Hinge" model described in the paper, other models were also used

to create ensembles. The "IDP" model used a flexible bead model for the linker and NTD, similar to that used in CORAL (Figure S4). The "SB" model mimics cAMP binding placing the flexible fitting region between the NBD and REM domains, residues 347-352, which is the region that melts upon cAMP binding. The "3-body" model has two flexible regions, from both the Apo and "SB" models. The "Camp" model is the (3CF6) cAMP bound homology structure with a tethered NTD, similar to the "Apo" (Linker: residues 80-94). The "Hinge-#" models use all of the extended conformations from "SB" and add the tethered NTD, for a compact well-sampled distribution (Figure 2).

Table S2. The apo-EPAC1 EOM distributions with alternative Models (0.5 mg/ml sample). The distributions based on the flexible NTD (aa 80-94), the melted hinge and switchboard "SB" (aa 348-351), or both "3-body". The results are binned into the percent fraction in each conformation: ($EPAC1_{closed}$) apo-EPAC1, an intermediate R_g range, ($EPAC1_{extended}$) the extended or cAMP-bound-like conformation. Each Entry list the R_g (Å), [peak width], and percent fraction (Figure S5). The intermediate R_g range is associated with aggregation or the lack of extended models.

Mutant	$EPAC1_{closed}$	Int-R_g	$EPAC1_{extended}$	X^2
**Apo**	33.0[0.5] 55%	36.6[0.7] 44%	NA	1.1
**IDP-NTD**	33.0[0.8] 86%	36.0[0.8] 12%	NA	1.1
**Apo + cAMP**	33.0[0.7] 87%	0%	42.0[0.8] 12%	1.0
**SB**	32.9[1.2] 83%	36.5[3] 6%	39.9[2.8] 10%	1.0
**SB + Apo**	32.9[0.5] 87%	0%	40.4[2.7] 12%	1.0
**3-body**	33.2[2.] 79%	35.4[3.] 14%	39.9[5.] 6%	1.0
**3-body + Apo**	33.1[2.] 79%	35.5[3.] 13%	40.6[6.] 7%	1.0
**Hinge-1 + Apo**	32.9[0.8] 86%	0%	38.5[0.9] 13%	1.0
**Hinge-3 + Apo**	32.9[0.8] 87%	0%	38.7[0.9] 11%	1.0
**Hinge-4 + Apo**	32.9[0.8] 86%	0%	37.5[0.9] 12%	1.0

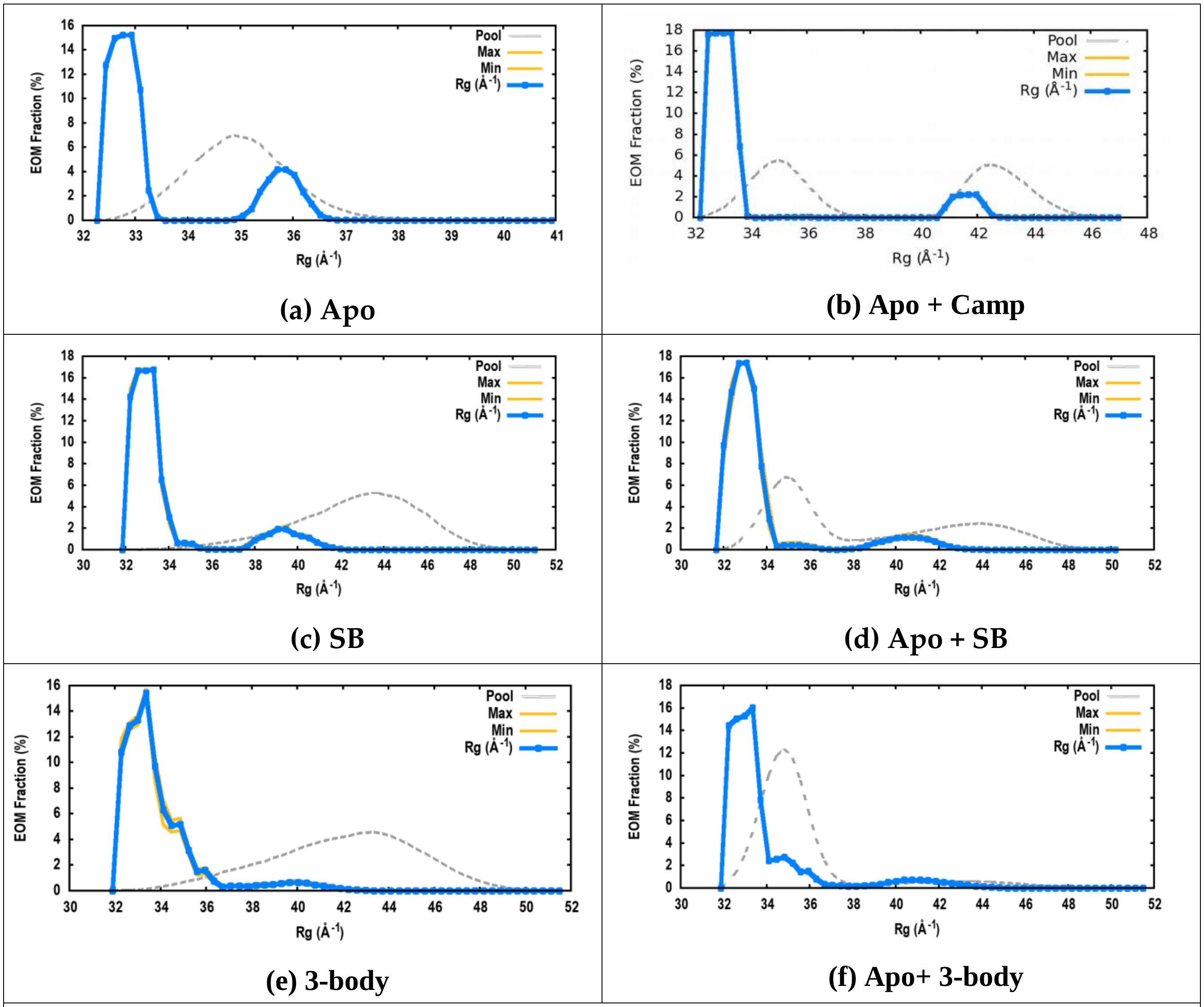

Figure S4: Alternate EOM Model R_g Distributions. (a) The apo-EPAC1 (EPAC1$_{open}$) mobile NTD "Apo" ensemble. The EOM R_g distribution is a blue line, the pool of "Apo" models a dashed grey line. The second peak is an artifact of an artificially limited range of models. (b) The apo-EPAC1 combined "Apo" and "Camp" ensembles. The two observed peaks are: 87% closed (R_g = 33.0 Å, FWHM 1.4 Å), and 12% extended (R_g = 42.0 Å, FWHM 1.6 Å). This distribution does not sample the 37-40 Å R_g range observed in the "SB" and "Hinge", and is model-limited. (c) The "SB" EOM distribution, based on the melted-hinge and switchboard (residues 347-351) modeled as a flexible linker between the regulatory N+DEP+CNDB domains and the catalytic REM+RA+GEF domains. The two observed peaks are: 90% closed (R_g = 35 Å, FWHM 1.2 Å), and 11% extended (R_g = 43 Å, FWHM 3. Å). (d) Adding the apo-EPAC1 conformations to the distribution pool results in a better fit: The two observed peaks are: 90% closed (R_g = 33 Å, FWHM 0.5 Å), and 11% extended (R_g = 40 Å, FWHM 2.7 Å). These second peak is still very broad. (e) A 3-body model, adds an additional degree of freedom to the "SB" model, by letting the NTD position vary simultaneously. The three observed peaks are: 79% closed (R_g = 33 Å), 14% aggregate (R_g = 35 Å), and 6% extended (R_g = 40 Å). (f) Adding the EPAC1$_{open}$ pool improves the distribution slightly. The three-body model has too many degrees of freedom which results in under sampling and broadening of the distribution peaks.

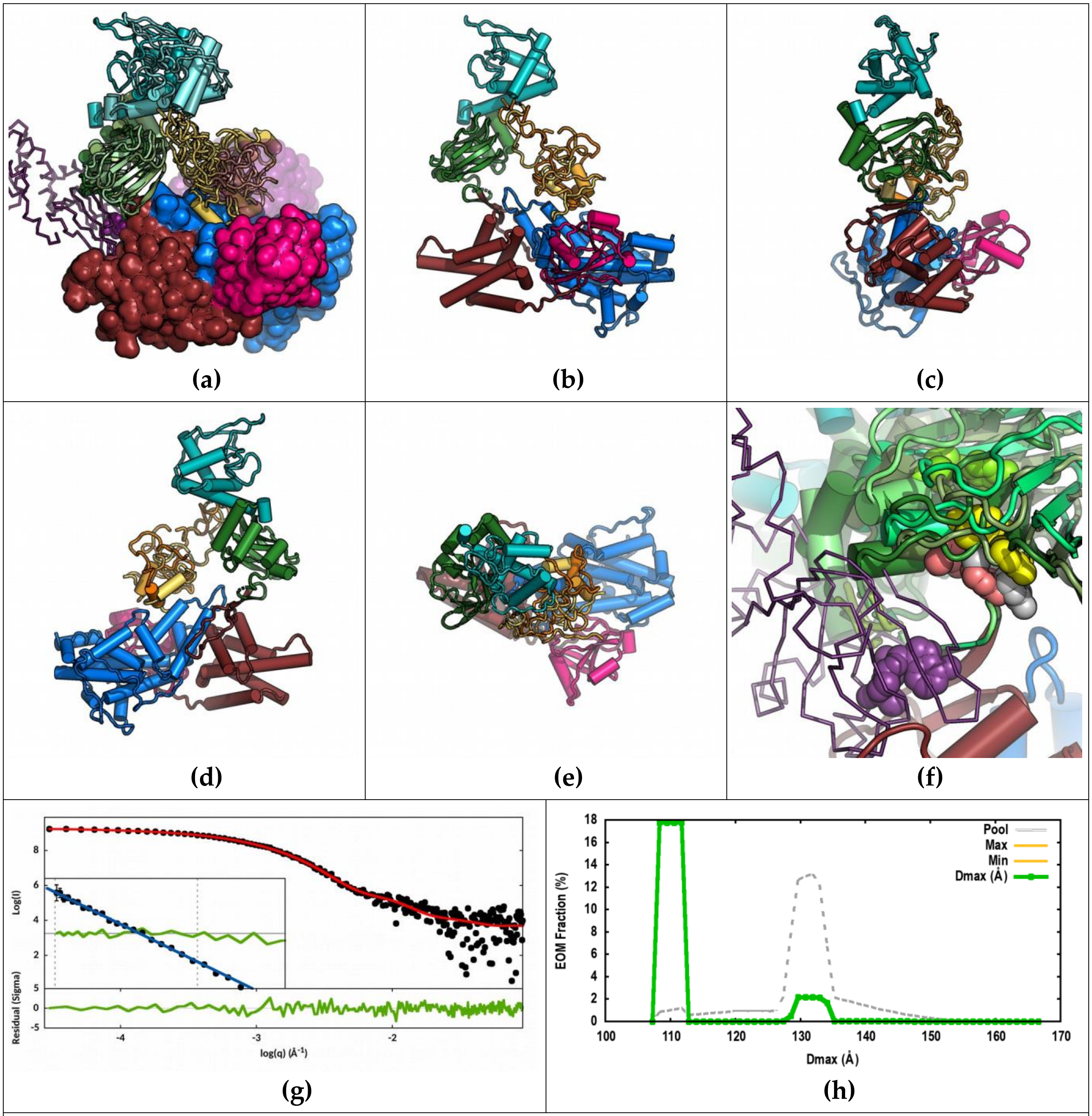

Figure S5. EOM Hinge and Switchboard "Hinge" Ensemble Models. The EOM apo-EPAC1$_{\text{extended}}$ models ("Apo" + "SB" pools) with a melted Hinge (Rg = 38.5 Å, 13%). The Models are coloured as in Figure 1. The ternary EPAC1 model is shown as a purple ribbon, the RAP a translucent surface. (a) Side view of all separately selected Hinge models and the ternary model. (b) Side view of the best ensemble. (c) End-on view of b. (d) View from opposite side to b. (e) Top view of b. (f) A close-up of the cAMP binding sites in the ternary (purple) and four Hinge templates (EPAC1$_{\text{extended}}$) models with cAMP marking their empty binding-sites (cAMP: green, yellow, pink, and gray). The cAMP moieties are in CPK coloured by model, and were added to show the cAMP binding-pocket in the Hinge models and in the ternary model (purple) where the SB's lid has closed over the cAMP. See Table S2 and Figure

2 for details. (g) The Log-Log plot of the EOM fit to the apo-EPAC1 data, $\chi^2 = 1.0$. (b) The Size (D_{max}) distribution plot.

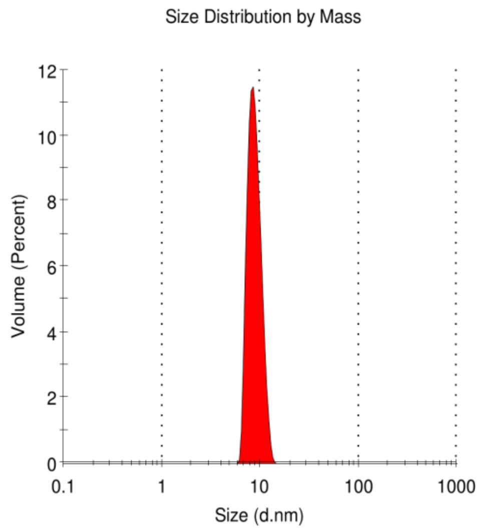

(a)

Figure S6: DLS analysis of apo-EPAC1 Sample. (a) Malvern Zetasizer Results: Peak-1 mode: 9.3 ± 1.4 nm; Pd: 15.2%; Est. MW 124 ± 19 kDa; Intensity: 100%; Mass: 100%; Monodisperse. No other peaks observed.
